# Estimation of the water content of needles under stress by *Erannis jacobsoni* Djak. via Sentinel-2 satellite remote sensing

**DOI:** 10.3389/fpls.2025.1540604

**Published:** 2025-04-15

**Authors:** Jiaze Guo, Xiaojun Huang, Debao Zhou, Junsheng Zhang, Gang Bao, Siqin Tong, Yuhai Bao, Dashzebeg Ganbat, Dorjsuren Altanchimeg, Davaadorj Enkhnasan, Mungunkhuyag Ariunaa

**Affiliations:** ^1^ College of Geographical Science, Inner Mongolia Normal University, Hohhot, China; ^2^ Inner Mongolia Key Laboratory of Remote Sensing & Geography Information System, Inner Mongolia Normal University, Hohhot, China; ^3^ Inner Mongolia Key Laboratory of Disaster and Ecological Security on the Mongolia Plateau, Inner Mongolia Normal University, Hohhot, China; ^4^ Forest Biological Disaster Prevention and Control Station, Yakeshi, Inner Mongolia, China; ^5^ Institute of Geography and Geoecology, Mongolian Academy of Sciences, Ulaanbaatar, Mongolia; ^6^ Institute of Biology, Mongolian Academy of Sciences, Ulaanbaatar, Mongolia

**Keywords:** *Erannis jacobsoni* Djak., leaf weight content dry, leaf weight content fresh, vegetation index, remote sense

## Abstract

**Introduction:**

*Erannis jacobsoni* Djak.(EJD) is one of the major pests that severely threatens forest health. Its damage predominantly affects pine species, resulting in significant changes to the biochemical composition of needle leaves. Needle leaf water content exhibits a clear response to these changes and is highly sensitive in reflecting the degree of tree damage.

**Methods:**

In this work, we combine vegetation indices with machine learning algorithms to estimate the water content of needles at a large scale. Multiple vegetation indices are screened via recursive feature elimination cross validation (RFECV), and then support vector regression (SVR) and back-propagation neural network (BP) models are used to predict the leaf weight content fresh (LWCF) and leaf weight content dry (LWCD) of needles over a large area. The water content ranges were then classified based on the severity of damage derived from actual sampling data. These ranges were used to categorize the estimated water content, thereby assessing the degree of tree damage. The accuracy of the method is verified by comparing the estimation results with field measurements, and the results are combined with the classifications of the leaf loss rate(LLR) to assess the severity of infestation.

**Results:**

The results indicate that: 1) When estimating LWCD and LWCF using the SVR and BP models, the SVR model demonstrated superior accuracy and stability (MAE for LWCF = 0.1477, RMSE = 0.17314; MAE for LWCD = 0.10507, RMSE = 0.14760). 2) The classification accuracies of LWCD and LWCF were notably higher in areas with light and medium damage, suggesting that these indices are effective indicators for assessing damage caused by *Erannis jacobsoni* Djak. and can serve as valuable tools for monitoring pest infestation and its progression. 3) Through precision evaluation and supplementary validation, the results show that LWCD is more stable and reliable than LWCF, demonstrating greater credibility, particularly in terms of MAE and RMSE, where LWCD exhibits lower values (MAE for LWCD = 0.10507, RMSE = 0.14760). This method’s high reliability provides an effective approach for estimating leaf weight content, both fresh and dry (LWCF and LWCD), and underscores its significant potential for the early monitoring and management of forest pests.

## 1 Introduction

Mongolia is dominated by mountainous terrain, and its geological structure is complex and varied, encompassing a wide range of typical ecosystem types such as alpine grasslands, primary forests, steppes, and deserts. Among them, primary forests, which are mainly composed of larch, account for 72% of the country’s total forest area. In recent years, forested areas in the northern and northeastern parts of the Mongolian Plateau have experienced large-scale infestations by coniferous insects, especially *Erannis jacobsoni* Djak.(EJD) (Bai et al., 2024; Xi et al., 2021). This pest causes significant damage to forest ecosystems, and consecutive years of infestation can result in the death of large areas of larch trees. The needle water content has become a central monitoring indicator of the response to this pest (Quemada et al., 2021; Tian et al., 2022; Song et al., 2023). Abnormal reductions in needle moisture content are often early indicators of the onset of infestation activity. Monitoring this indicator not only serves as an important early warning of pest presence but also helps researchers recognize signs of pest damage in a timely manner. This timely monitoring and early warning mechanism can be effective in initiating necessary interventions to mitigate the long-term impacts of infestations on forests. Given the threat to forest ecosystems posed by EJD, early monitoring and prevention are particularly important and urgently needed.

Traditional monitoring of forest pests and diseases is usually based on ground surveys, but ground surveys are often difficult due to limitations associated with complex terrain, lack of transportation, and high human and material costs (Cornelius et al., 2017). These methods are time-consuming, costly, and inefficient over large areas of forest. In recent years, with the rapid development of remote sensing technology, a variety of remote sensing images have been widely used for monitoring forest pests and diseases (Park et al., 2023; Dottavio and Williams, 2024). Remote sensing technology can cover a wide area and provide continuous monitoring data with high spatial and temporal resolutions, which greatly reduces the costs of traditional monitoring methods (Zhang et al., 2022; Decuyper et al., 2022). In this context, the vegetation index, as an important parameter in remote sensing technology, can be used to indirectly assess the health of vegetation and has become an important indicator for forest health monitoring (Huete, 2012; Zhang Y. et al., 2024). Researchers have employed vegetation indices to assess the severity of insect damage (Marx et al., 2024; Adan et al., 2023), detect signals of forest degradation (Lasaponara et al., 2024), and identify early indicators of pest infestations (Tang et al., 2023; Zhang S. et al., 2024). Furthermore, other scholars have used vegetation indices to estimate physiological parameters such as chlorophyll content (Li et al., 2023; Sun et al., 2021) and moisture levels (Zhang et al., 2019) in plants, achieving promising results. In conclusion, the application of vegetation indices as tools for the indirect monitoring of vegetation health in several regions of the world has proven effective. This multi-index approach not only enhances the reliability of monitoring data but also provides valuable information resources for scientific management and ecological conservation.

Moreover, in the remote sensing monitoring of vegetation pests and diseases, machine learning techniques demonstrate considerable potential, particularly in data analysis and model prediction (Narmilan et al., 2022; Guo et al., 2024; Waters et al., 2025). Research indicates that utilizing machine learning methods to identify and predict pest risk across various plant species can effectively reduce human errors and enhance operational efficiency (Bai et al., 2014; Ahmad et al., 2021; Chithambarathanu and Jeyakumar, 2023; Lee and Yun, 2024). These techniques can be combined with vegetation index data obtained via remote sensing at a large scale to achieve efficient and accurate pest monitoring.

Early monitoring of infestations is a challenge in forest management, mainly because infestations are often difficult to detect in the early stages through traditional ground surveys (Rahimzadeh-Bajgiran et al., 2018; Bhattarai et al., 2023). This monitoring difficulty not only delays the optimal time for pest control but also leads to the rapid spread of pests and severe damage to forest ecosystems, issues that must be urgently addressed. The monitoring of the water content of needles and leaves provides an effective solution for the early detection of insect pests, especially with the support of remote sensing technology (Curran, 1989; Villacrés et al., 2021); notably, we are able to extract a variety of vegetation indices closely related to plant physiological status by analyzing spectral features. Since individual vegetation indices may display inconsistent trends in different environments, the effective selection and combination of indices are the keys to improving the accuracy of conifer water content inversion (Ihuoma and Madramootoo, 2019; Tan et al., 2019). On this basis, this study aims to provide an efficient and reliable method to select sensitive indices from multiple vegetation indices that effectively reflect changes in the plant water content to improve the accuracy of conifer water content estimation, which can be used to rapidly estimate the conifer water content over a wide range of areas damaged by the EJD. Moreover, the results provide an experimental basis for the monitoring and management of forests in the early stages of infestation and stress.

## 2 Research concepts and research areas

### 2.1 Research concepts

**Figure 1:**
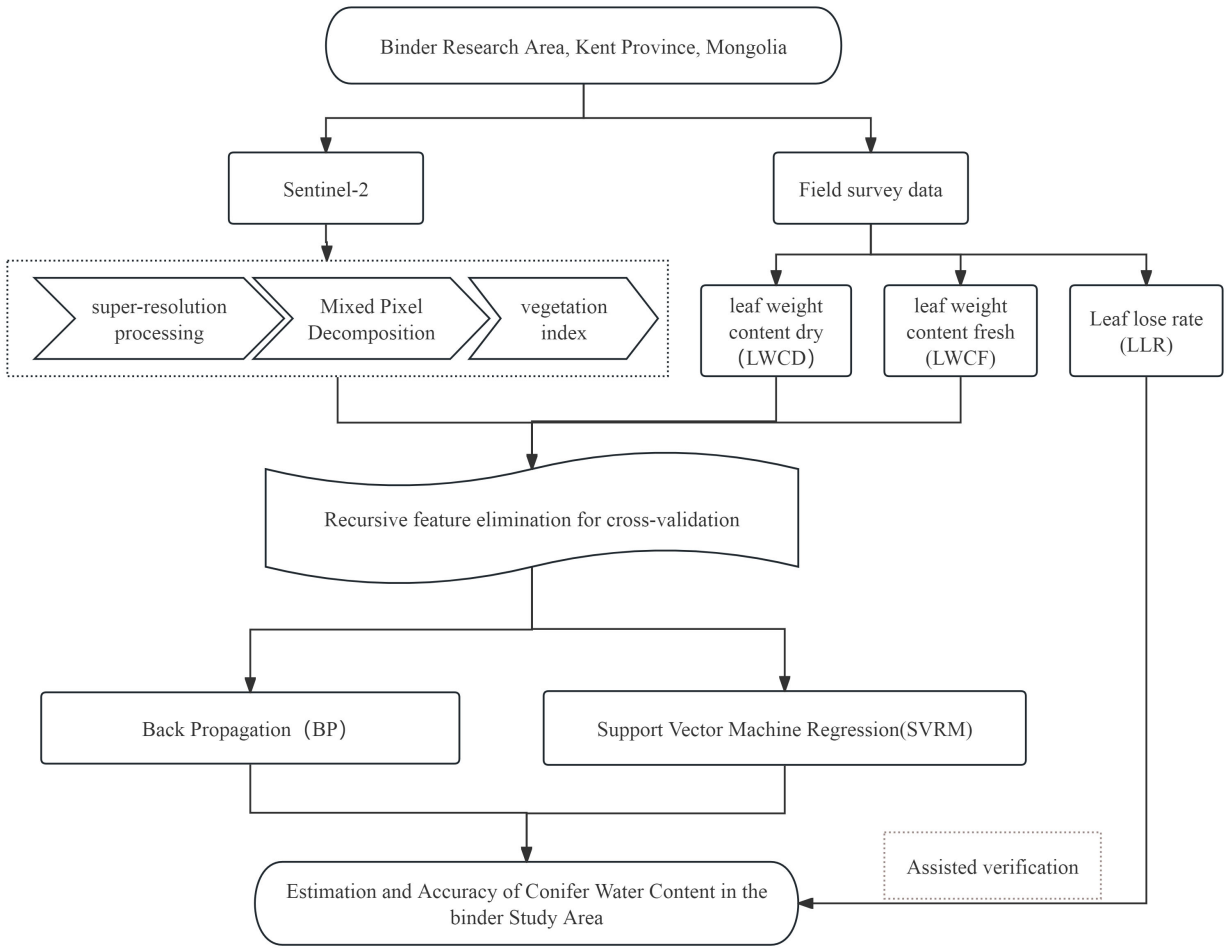
Technical roadmap.

This study combines drone and Sentinel-2 satellite imagery to propose an overall research methodology for estimating needle leaf water content under EJD stress (**Figure 1**).

### 2.2 Study area

#### 2.2.1 Overview of the study area

**Figure 2:**
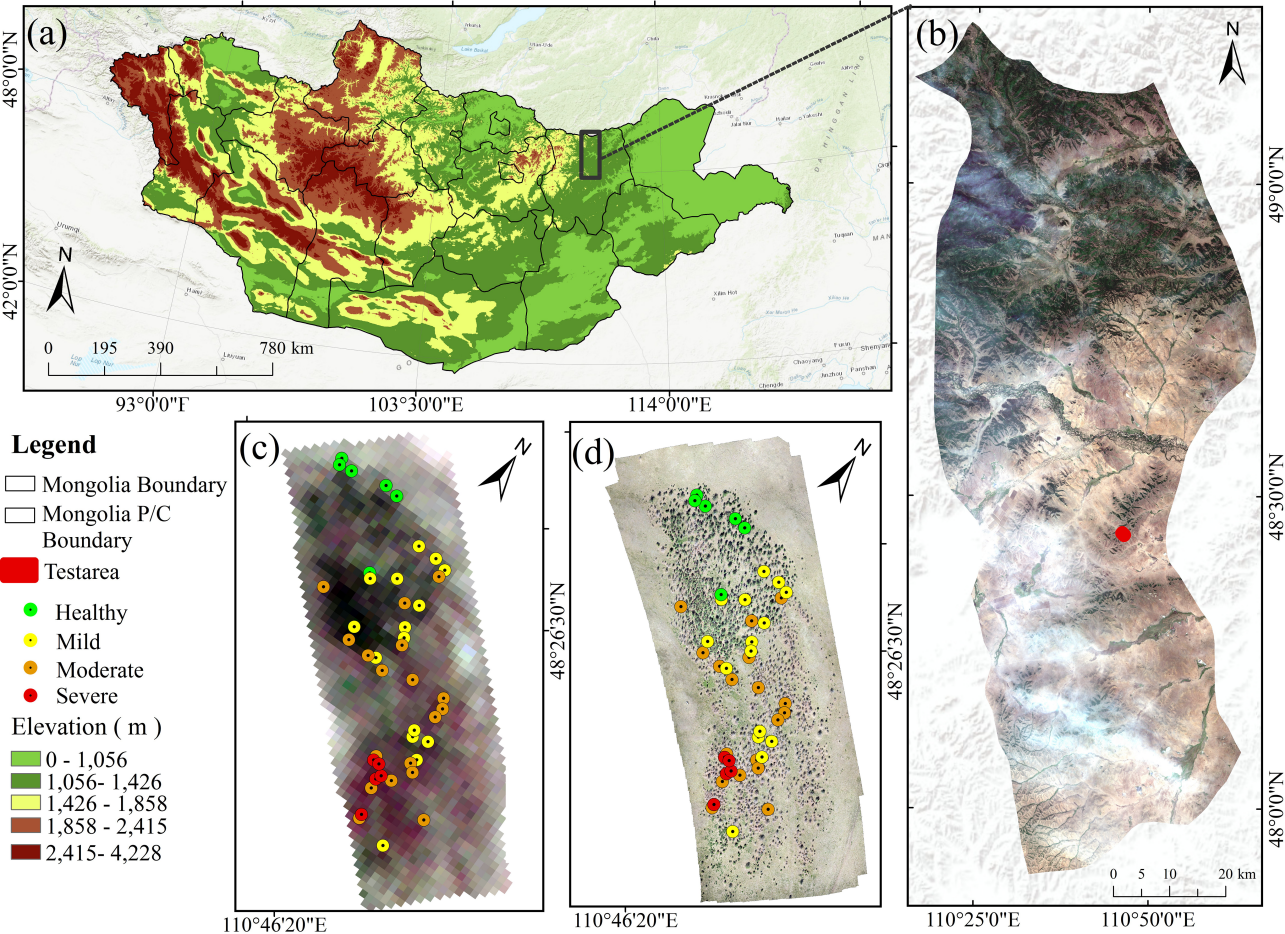
Overview map of the study area: **(a)** topographic map of Mongolia; **(b)** Binder region; **(c)** remote sensing image of the study area; **(d)** UAV image of the study area.

The present study was conducted in the outbreak area of EJD in Binder (longitude 110°46'33.6''E~110°46’33.6’’E, latitude 48°26'34.8''N~48°26’34.8’’N), Kent Province, Mongolia (**Figure 2**). The area is characterized by complex geomorphic features, such as mountains, forests, grasslands, and river valleys. The Binder area is known for its high elevation and undulating terrain, and the vegetation is dominated by larch, forming a typical coniferous forest ecosystem. Climatically, the region has a temperate continental climate, with long, cold winters and short, wet summers. The annual precipitation is relatively low, but localized areas receive significant rainfall in the summer. According to reports, the Binder area experienced consecutive large-scale outbreaks of EJD in 2017, 2018, and 2019, resulting in severe damage to local larch forests. The needles of many trees were eaten by the pest, which severely affected the stability of the forest ecosystem. Owing to the periodic outbreaks of this pest, the Binder region has become a key area of concern for domestic and international researchers, prompting further strengthening of research on forest health monitoring and pest control. In particular, during the 2019 outbreak, the pest destroyed many needles and leaves, significantly reduced photosynthesis in trees, and impaired physiological functions, resulting in the death of some trees. The infestation not only had far-reaching impacts on forest ecosystems but also posed significant challenges to the regional economy and forestry management, highlighting the region’s vulnerability to pests. Some studies have shown that the leaf loss rate (LLR) is an effective indicator of tree damage, which can directly reflect the impact of an insect infestation on tree health (Češljar et al., 2022). Building on this, the present study calculated the LLR through field surveys and categorized the severity of damage into four levels based on the LLR: 0%–5% as healthy; 6%–30% as lightly damaged; 31%–70% as moderately damaged; and 71%–100% as severely damaged. This classification follows the methodology established in our previous work (see Huang et al., 2019).

#### 2.2.2 Ground truth and survey data

The lifecycle of EJD begins with an incubation period from late April to mid-May, followed by a larval stage from late May to early July, when the larvae cause severe damage to vegetation. This stage is followed by the pupal stage from mid-July to early September, followed by the plumage stage from early September to mid-October, when the adults mate and lay eggs. Finally, from late October to April, the eggs enter the overwintering period until they hatch the following year (Li et al., 2023). In this study, drone images were taken in the test area in June 2019, during the larval stage. In total, 44 sample plots measuring 10 m × 10 m were selected within the test area on the basis of the similarity of stand severity to provide data support for the remote sensing monitoring of EJD infestations.

**Figure 3:**
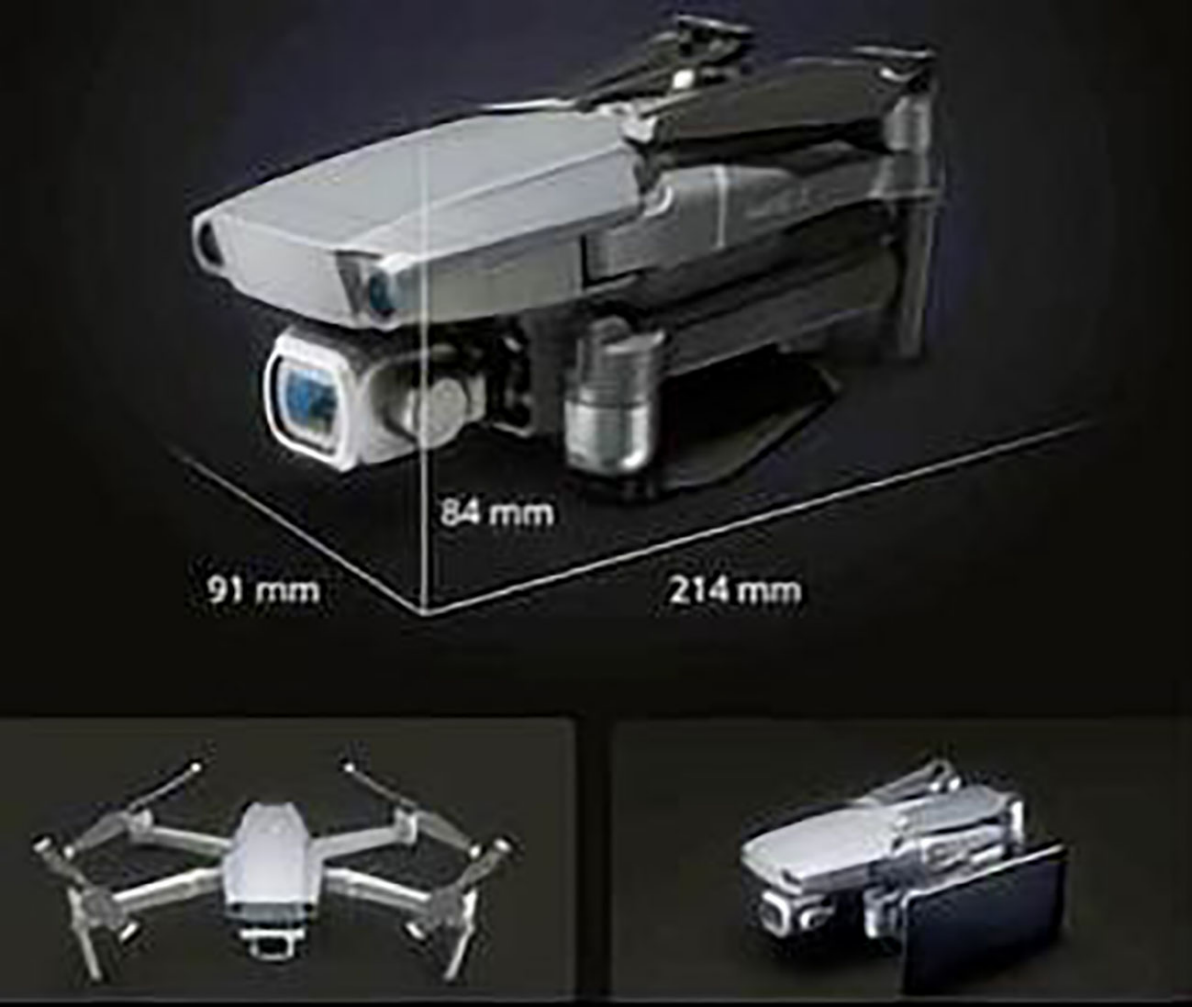
DJI “Mavic 2” drone.

A DJI Mavic 2 professional drone was used to take aerial photographs of the Binder test area (**Figure 3**), acquiring RGB image data with a spatial resolution of 0.02 m. The Mavic 2 drone can fly for 31 minutes, and its Hasselblad L1D-20c camera is capable of taking nine medium-focus photographs with automatic stabilization, from which ultra high-resolution images with 4x resolution and 48 megapixels can be produced. Cloud-free, rain-free, and wind-free conditions were chosen for operations during the image capture process. To meet the flight and image quality requirements, the unmanned aerial vehicle (UAV) flight altitude was set to 60 m, and the heading overlap and side-to-side overlap were 80% and 60%, respectively.

In each sample plot within the experimental area, five sample trees were uniformly selected, from which needles were collected and placed in a sealed plastic bag to prevent moisture loss. The fresh weight (FW) of each sample was subsequently measured via an electronic balance with an accuracy of 0.0001 grams. All the samples were dried in a drying oven at 80°C for 48 hours and then weighed again to record the dry weight (DW). The fresh leaf weight content (LWCF) and dry leaf weight content (LWCD) of the needles were calculated according to Equations 1 and 2.


(1) $$\text{LWCF}=\frac{\text{FW}-\text{DW}}{\text{FW}}\times 100\%$$

(2) $$\text{LWCD}=\frac{\text{FW}-\text{DW}}{\text{DW}}\times 100\%$$

To assess the severity of pine caterpillar infestation, this study employs the LLR for classification. LLR is defined as the ratio of leaf loss to the total number of leaves per unit area of the tree crown (Huang et al., 2019). During the calculation, typical standard branches were selected from five levels of the sample trees (upper, upper-middle, middle, middle-lower, and lower), with one branch chosen from each of the four cardinal directions (east, south, west, and north). The number of affected and healthy needles was recorded for each direction. The average LLR for all branches was then calculated using Equation 3, yielding the LLR for the sample tree. The mean LLR of the five sample trees was taken as the LLR for the plot. This methodology ensures a thorough and accurate assessment of infestation severity. In the formula, LLR represents the LLR for the branch, while N_i_ and N_h_ denote the counts of healthy and lost needles, respectively.


(3) $$\text{LLR}=\frac{{\text{N}}_{i}}{{\text{N}}_{\text{h}}+{\text{N}}_{\text{i}}}\times 100\%$$

### 2.3 Remote sensing image data

The Sentinel-2 series of satellites was launched as part of an Earth observation mission under the Copernicus program of the European Space Agency. The satellites are mainly used for periodic high-resolution imaging to monitor forest vegetation, land cover, soil moisture, water resources, and natural disasters on the Earth’s surface. The series consists of two satellites, Sentinel-2A and Sentinel-2B, with a revisit period of 10 days for a single satellite and a joint revisit period of 5 days when the two satellites are working in combination, especially at high latitudes in Europe, where the revisit period can reach 3 days. The Sentinel-2 satellites carry a multispectral imager with 13 spectral bands; however, the spatial resolution of these bands varies, including four spectral bands with different spatial resolutions, which are used for the monitoring of forest cover, soil moisture, water resources, and natural disasters. Sentinel-2 includes multispectral imagers with 13 spectral bands; however, the spatial resolution of these bands varies, with four bands having a spatial resolution of 10 m, six bands with a spatial resolution of 20 m, and three bands with a spatial resolution of 60 m. Users can download these satellite images free of charge from the official Copernicus website. The specific band information is detailed in **Table 1**. Notably, the satellite has three bands in the near-infrared spectral region, which is the only multispectral optical data source that covers three bands in the red-edge range. Therefore, Sentinel-2 images have significant advantages over other images in fields such as vegetation health monitoring, crop growth assessment, and chlorophyll concentration measurement in water bodies.

**Table 1:** Sentinel-2 spectral bands.

Band	Resolution	Central wavelength	Primary application	Band used in this study
B1	60m	443nm	Coastline	
B2	10m	490nm	Blue light	✓
B3	10m	560nm	Green light	✓
B4	10m	665nm	Red light	✓
B5	20m	705nm	Vegetation red edge	✓
B6	20m	740nm	Vegetation red edge	
B7	20m	783nm	Vegetation red edge	
B8	10m	842nm	Near infrared	✓
B8a	20m	865nm	Vegetation red edge	✓
B9	60m	940nm	Water vapor	
B10	60m	1375nm	Shortwave infrared, cirrus clouds	
B11	20m	1610nm	Shortwave infrared	✓
B12	20m	2190nm	Shortwave infrared	

## 3 Research methods and data processing

### 3.1 Super-resolution processing of remote sensing images

This study selected two Sentinel-2 images acquired simultaneously with the drone observations taken on 21 June 2019. Both images are Level-2A data, with the image processing date being 16 October 2023. The images contained information from a total of 10 bands, i.e., B2, B3, B4, B5, B6, B7, B8, B8A, B11, and B12, with a spectral range covering 490 to 2190 nm. The 60-m resolution coastal aerosol (B1), water vapor (B9), and cirrus (B10) bands were excluded because they are not relevant to this study. Then, the two-scene images were mosaicked to ensure complete coverage of the study area, and the results were exported in TIF format for subsequent analysis. During image analysis, the image elements were first classified according to the degree of damage: (1) healthy image elements, which mainly included healthy larch; (2) mildly damaged image elements; (3) moderately damaged image elements; and (4) severely damaged image elements, which mainly consisted of severely damaged or dead larch.

To ensure the consistency and accuracy of the data in different bands, the processing of remote sensing images was carried out via SNAP 6.0 software. Given the different spatial resolutions of the bands of Sentinel-2, the super-resolution enhancement technique was used in SNAP to upgrade the 20-meter resolution band to 10 meters. This processing step was critical because it ensured consistent spatial resolution across all bands, thereby improving the accuracy and reliability of subsequent analyses. Super-resolution enhancement is a technique via which one high-resolution image is generated by fusing multiple low-resolution remote sensing images. In this method, the spatial details of the image are first refined through complex algorithms and interpolation techniques to extract geometric information from high-resolution bands that are independent of the spectral bands while preserving the reflectance information from each low-resolution band. A hybrid model is constructed to downsample the data from the high-resolution bands to a consistent low resolution and optimize the shared values and weights by minimizing the differences between the high-resolution data and the downsampled reconstructed data. After the shared values and weights for the high-resolution bands were obtained, this information was applied to the low-resolution bands to generate super-resolution images. Super-resolution synthesis is widely used in the fields of environmental monitoring, urban planning, and resource management and can significantly increase the application value and analytical capability of remote sensing data.

### 3.2 Mixed-pixel decomposition

In recent years, with the rapid development of remote sensing technology, remote sensing images have been widely used in the fields of feature information acquisition and environmental monitoring. However, owing to the limitations associated with spatial resolution and complex feature distributions, the mixed-pixel phenomenon often occurs in remote sensing images, i.e., one pixel may contain spectral information for multiple feature types (e.g., buildings, vegetation, and water bodies). To address this problem, researchers have proposed different mixed-image-element decomposition models, which are mainly categorized into linear and non-linear methods (Gao et al., 2020). The linear hybrid image element decomposition model consists of two parts: end-element extraction and abundance inversion. End-element extraction is the key step, which directly affects the accuracy of the decomposition results. The end elements represent the spectral features of pure elements in the image, and after the end elements are extracted, the spectral information from the hybrid image can be decomposed to determine the abundance of different feature types. The accuracy of end-element extraction crucially influences abundance inversion.

**Figure 4:**
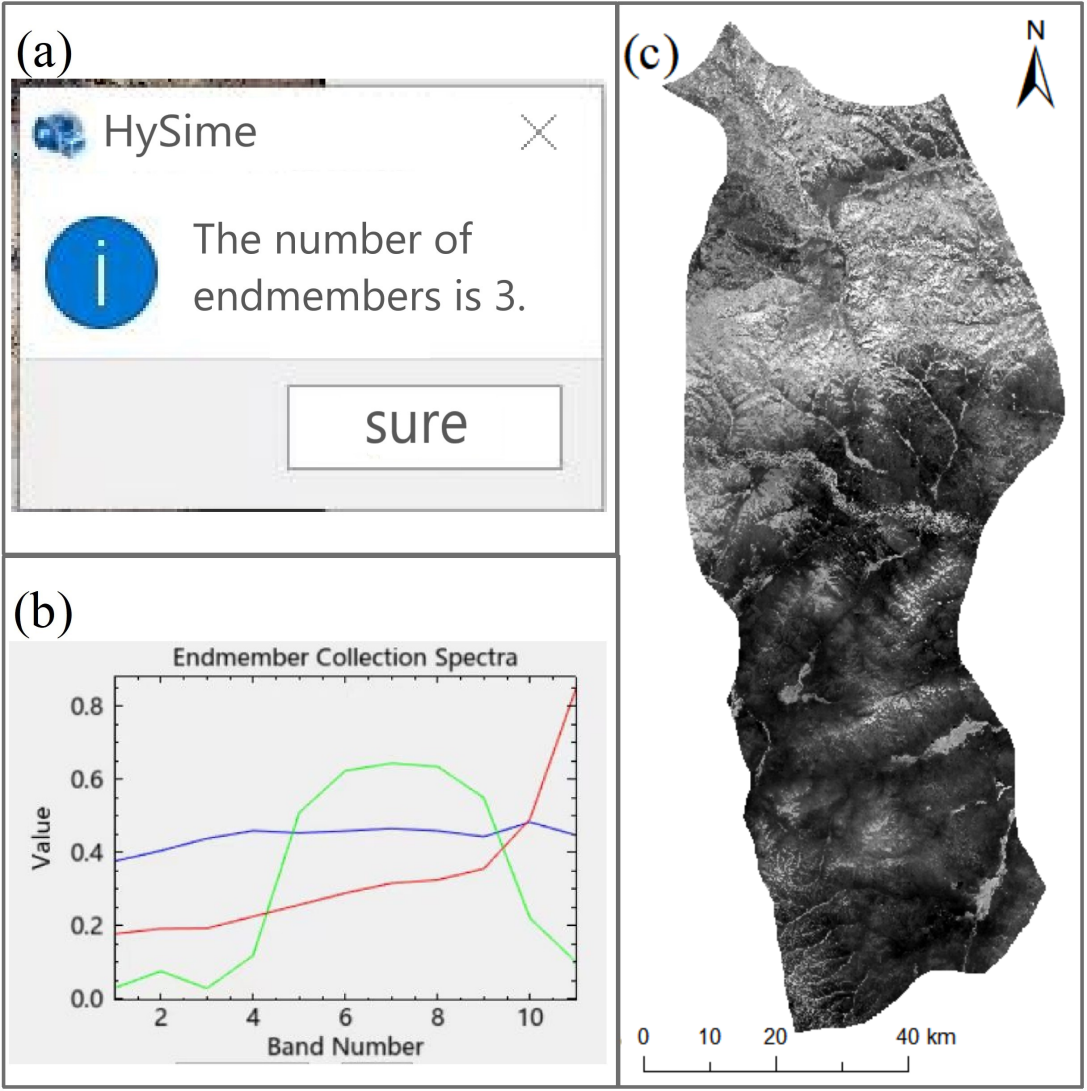
HySime algorithm analysis: **(a)** Use of the HySime algorithm to estimate the number of end elements, with three identified; **(b)** spectral signature curve of extracted end elements; **(c)** extracted vegetation information for pure end elements.

In this paper, the HySime (hyperspectral signal identification by minimum error) algorithm, a hyperspectral subspace identification algorithm that was proposed by José M. et al., is used for end-element extraction, implemented using Python. The algorithm obtains the effective bands after hyperspectral dimensionality reduction through a subspace identification step, which is an important preprocessing step in processing algorithms such as target detection, change detection, classification, and hybrid image element decomposition and helps improve the storage and reduce the complexity of hyperspectral data. On the basis of the computational results of the HySime algorithm, three end elements are identified in this paper (shown in **Figure 4**). The spectral features of these end elements are used as a reference for subsequent hybrid image element decomposition and hyperspectral classification, similar to the samples in supervised classification, which highly influence the accuracy of the decomposition results. The core role of end-element extraction is to obtain the spectral features of pure elements from a hyperspectral image, which directly influences the accuracy of the abundance inversion. Then, the vertex component analysis (VCA) algorithm, which is based on the geometric form of the linear spectral mixing model, is constructed, and the end-element spectra are extracted step by step on the basis of orthogonal vectors. Next, the projections of the image matrices are obtained on the basis of these orthogonal vectors to provide support for subsequent hyperspectral analysis.

In the abundance inversion step, a fully constrained least squares (FCLS) approach is applied for hybrid image element decomposition; this is a remote sensing image processing technique based on statistical methods. In hybrid image element decomposition on the basis of remotely sensed images, each pixel is treated as a hybrid image element consisting of multiple features, and the contribution of each feature in the image element is estimated from the image data. The FCLS method is used in this decomposition process, and a system of linear equations is solved under specific constraints.

### 3.3 Multispectral vegetation index calculation

During the process by which larch is subjected to insect pest stress, internal biochemical components, such as the needle water content, significantly change with increasing pest severity, resulting in different canopy reflectance responses. On the basis of this phenomenon, the multispectral vegetation index (MVI) was selected as an index for estimating the LWC in this study. Specifically, 40 vegetation indices, including the blue, green, red, red-edge, near-infrared, and other bands, were calculated via ArcGIS software (Gitelson and Merzlyak, 1994; Rondeaux et al., 1996; Brodu, 2016; Chrysafis et al., 2020; Ma, 2023) (see **Table 2** for details), and the average vegetation index values of the blended image elements were extracted. This process provided the necessary database for the subsequent analysis and helped reveal the relationships between the vegetation indices and conifer water content.

**Table 2:** Vegetation indices and corresponding formulas.

Number	Vegetation index	Formula
1	Reflectance index 2 (ARI2)	(B8/B3)−(B8/B5)
2	Chlorophyll concentration reflectance index (CCRI)	B4/B5
3	Chlorophyll red edge index 1 (CRI1)	(1/B2)−(1/B3)
4	Corrected transformed vegetation index (CTVI)	$\left(\frac{\text{NDVI}+0.5}{|\text{NDVI}+0.5|}\right)\times \sqrt{\left|\text{NDVI}+0.5\right|}$
5	Simple ratio 550/680 disease-water stress index 4 (DSWI-4)	B3/B4
6	Enhanced vegetation index (EVI)	2.5×(B8−B4)/(B8+6×B4−7.5×B2+1)
7	Enhanced vegetation index 2 (EVI2)	2.5×(B8−B4)/(B8+2.4×B4+1)
8	Green normalized difference vegetation index (GNDVI)	(B8−B3)/(B8+B3)
9	Modifies non-linear vegetation index (MNLI)	$1.5\left(\text{B}8^{0.5}-\text{B}4\right)/\left(\text{B}8^{0.5}+\text{B}4+0.5\right)$
10	Modified simple ratio - red edge (MSRreg)	$\left(\text{B}8/\text{B}5-1\right)/\sqrt{\left(\text{B}8/\text{B}5+1\right)}$
11	Normalized difference green index (NDGI)	(B3−B4)/(B3+B4)
12	Normalized difference infrared index 45 (NDI45)	(B5−B4)/(B5+B4)
13	Normalized difference infrared index (NDII)	(B8−B12)/(B8+B12)
14	Normalized difference moisture index (NDMI)	(B8−B11)/(B8+B11)
15	Normalized difference vegetation index (NDVI)	(B8−B4)/(B8+B4)
16	Normalized difference red edge index (NDREI)	(B8−B6)/(B8+B6)
17	Normalized difference vegetation index-red edge (NDVIreg)	(B8−B5)/(B8+B5)
18	Non-linear vegetation index (NLI)	$\left(\text{B}8^{2}-\text{B}4\right)/\left(\text{B}8^{2}+\text{B}4\right)$
19	Optimized soil adjusted vegetation index (OSAVI)	(B8−B5)/(B8+B5+0.16)
20	Optimize soil-adjusted vegetation index-red edge (OSAVIreg)	(1+0.16)(B8−B4)/(B8+B4+0.16)
21	Pigment-specific simple ratio (PSSR)	B8/B4
22	Renormalized difference vegetation index-red edge (RDVIreg)	$\left(\text{B}8-\text{B}5\right)/\sqrt{\left(\text{B}8+\text{B}5\right)}$
23	Ratio vegetation index-red edge (RVIreg)	B8/B5
24	Soil-adjusted vegetation index (SAVI)	1.5×(B8−B4)/(B8+B4+0.5)
25	Normalized difference 860/1640 (SIWSI)	B8a−B11/B8a+B11
26	Vegetation growth cycle index (VGCI)	(B8−B4)/B4
27	Aerosol free vegetation index 1600(AFRI1600)	B8−0.66[B11/(B8+0.66×B11)]
28	Aerosol free vegetation index 2100(AFRI2100)	B8−0.5[B12/(B8+0.56×B12)]
29	Difference vegetation index (DVI)	B8−B4
30	Difference vegetation index (Reg.) (DVIreg)	B8−B5
31	Internal vegetation index 2 (Int2*)	(B3+B4+B5)/2
32	Simple index 1 (SI1*)	$\sqrt{\text{B}5\times \text{B}4}$
33	Generalized multispectral normalized difference vegetation index (GMNLI)	$1.5\left(\text{B}8^{0.5}-\text{B}3\right)/\left(\text{B}8^{0.5}+\text{B}3+0.5\right)$
34	Simple index (Reg.) (SIreg)	$\sqrt{\text{B}2+\text{B}5}$
35	Modified triangular vegetation index 2 (MTVI2)	$\frac{1.5\left[1.2\left(\text{B}8-\text{B}3\right)-2.5\left(\text{B}4-\text{B}3\right)\right]}{\sqrt{{\left(2\text{B}8+1\right)}^{2}-\left(6\text{B}8-5\text{B}4^{0.5}\right)-0.5}}$
36	Generalized difference vegetation index (GDVI)	B8−B3
37	Non-linear vegetation index 2 (NLI 2)	$\left(\text{B}8^{2}-\text{B}3\right)/\left(\text{B}8^{2}+\text{B}3\right)$
38	Chlorophyll index (Reg.) (CIreg)	B8/B5−1
39	Soil chlorophyll content index (SCCI)	100×(B8−B4)/NDVI
40	Red edge normalized difference vegetation index (red_edge_ndvi)	(B8−B5)/(B8+B5)

### 3.4 Sensitive feature selection

Selecting sensitive features for multispectral vegetation indices is crucial before modeling because using too many variables increases the computational time and complexity of the model and sometimes even reduces the accuracy of the model. In the fields of statistics and machine learning, recursive feature elimination with cross-validation (RFECV) is an efficient feature selection method that combines recursive feature elimination (RFE) with cross-validation (CV) (Awad, 2023) In this study, the RFECV method is used to optimize model performance by progressively eliminating the least important features and assessing the model accuracy at each step via cross-validation to determine the optimal number of retained features.

In RFE, the model is first trained using all available features, and then the least important features are progressively removed on the basis of the feature importance score provided by the model. The process is repeated, removing one or more of the lowest-scoring features at a time until a preset number of features is reached or a certain model performance criterion is met. RFECV extends this approach further by automatically determining the optimal number of features through cross-validation, independent of the preset number of features. In RFECV, the entire RFE process is repeated with different cross-validation folds. For each feature subset, the model is trained with a training set, and its performance is evaluated with a validation set, where the model performance after each feature elimination step is recorded, and the feature subset that results in the highest cross-validation score is finally obtained.

By introducing CV, RFECV is not only able to find the feature subset that performs best for the current data but also enhances model generalization ability by repeating the training and evaluation processes several times, thus reducing the risk of overfitting. This approach is particularly suitable for complex datasets with many highly correlated features, significantly improving the prediction accuracy and robustness of models.

### 3.5 Estimation models

In this study, two classical machine learning algorithms, back propagation (BP) and support vector regression (SVR), were used to model the water content of conifers, and the performance of the models was systematically compared.

The BP algorithm is a widely used training method in the field of artificial neural networks, particularly well-suited for multilayer neural networks, such as multilayer perceptrons. In the training of the BP algorithm, we employed Leave-One-Out Cross-Validation (LOOCV) to evaluate the model's performance (Silva et al., 2024). The LOOCV approach treats each data point as an individual test set while using the remaining data as the training set. After conducting multiple rounds of training and testing, the model’s average accuracy was calculated. To prevent overfitting, we set the number of iterations to 1,000 and implemented an early stopping strategy. Additionally, we selected a learning rate of 0.01, 40 nodes for the hidden layer, and applied standard gradient descent for optimization.

In contrast, support vector machine regression (SVMR) is a regression technique based on the principles of support vector machines (SVM). During the training of the SVMR model, we also utilized LOOCV and optimized the model parameters by selecting different training sets in each iteration. For the SVMR model, we employed the radial basis function (RBF) kernel, with the penalty parameter (C) set to 10 and the kernel parameter (γ) set to 0.1. These hyperparameters were fine-tuned through cross-validation to ensure strong generalization performance.

A key advantage of LOOCV is that it trains and tests the model multiple times, with each data point serving as the test set once. This allows for maximum utilization of the available data for training, minimizing potential biases introduced by data partitioning. Thus, LOOCV is particularly well-suited for small sample datasets, ensuring optimal data usage while providing a reliable evaluation of the model’s stability and generalization capability.

By employing this methodology, the performance of both the BP and SVMR models was thoroughly evaluated, and optimal hyperparameter configurations were identified. These models have been widely applied in the early detection and management of forest pests, playing a significant role in the protection of forest ecosystems. Through cross-validation, we have ensured the predictive accuracy of the models, providing a solid technical foundation for addressing similar challenges in future studies.

### 3.6 Evaluation of model accuracy

In this study, the root mean square error (RMSE) and mean absolute error (MAE) are selected as the evaluation metrics (Equations 4, 5), where the RMSE is a commonly used metric to evaluate regression models and quantifies the difference between the predicted values of the model and the actual observed values. It provides a measure of the error, and the smaller the error is, the better the performance of the model is in most cases. The MAE usually ranges from 0 to infinity, and it is a measure of the average of the absolute difference between the predicted and actual values. Compared with the RMSE, the MAE is less sensitive to outliers and, therefore, provides a more robust assessment of performance when outliers are present in the data.


(4) $$\text{MAE}=\frac{1}{\text{n}}\sum_{\text{i}=1}^{\text{n}}\left|{\text{y}}_{\text{i}}-\hat{{\text{y}}_{\text{i}}}\right|$$

(5) $$\text{RMSE}=\sqrt{\frac{1}{\text{n}}\sum_{\text{i}=1}^{n}{\left({\text{y}}_{\text{i}}-\hat{{\text{y}}_{\text{î}}}\right)}^{2}}$$

## 4 Results and analysis

### 4.1 Supervised classification of remote sensing data and forest cover extraction

**Figure 5:**
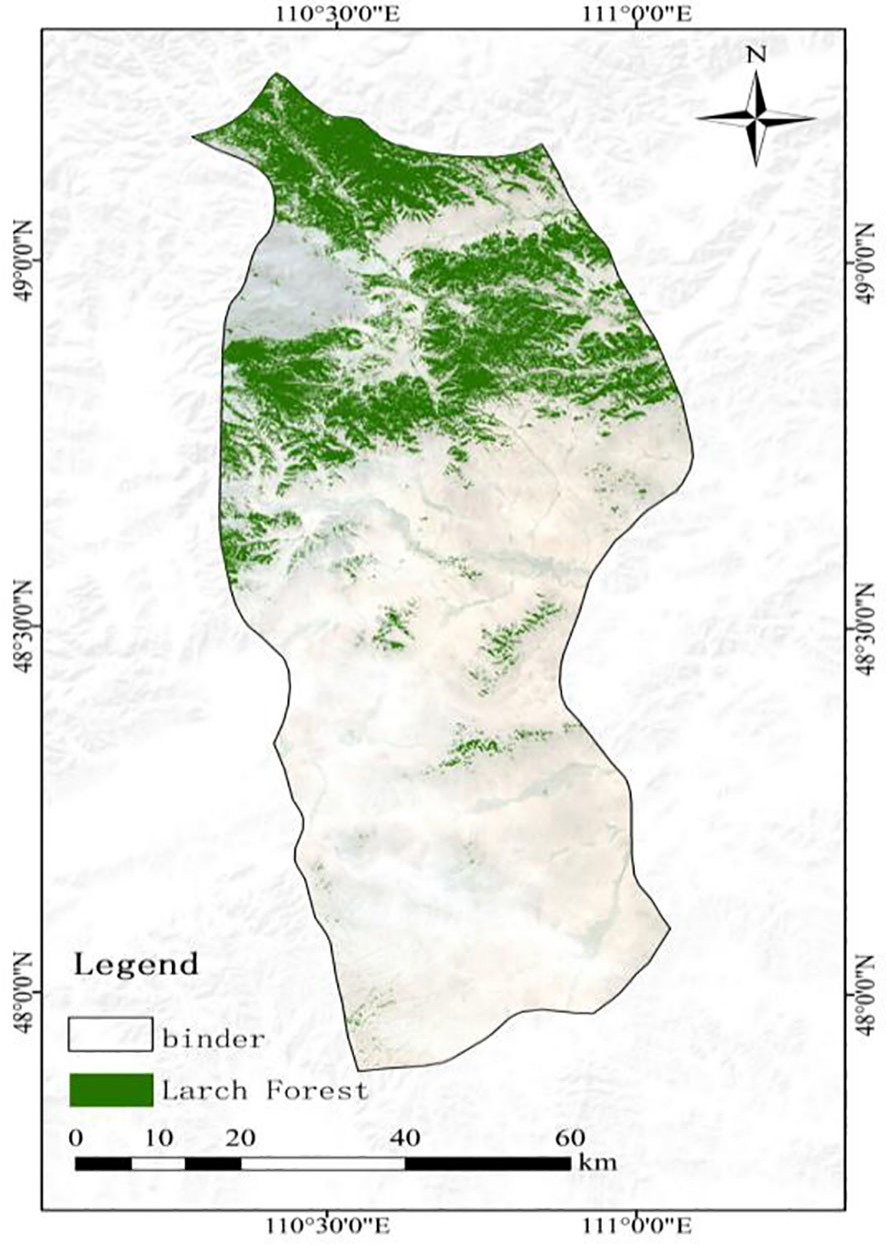
Forest data points extracted via supervised classification.

In this study, we used the maximum likelihood (MLC) method in ENVI software for the supervised classification of remotely sensed data in the Binder area. During the classification process, the study area was categorized into forests, water bodies, built-up areas, agricultural land, and bare land. Owing to the presence of cloud cover in the upper left corner of the study area, we removed this area from the images. To verify the accuracy of the classification results, we downloaded 2019 land use product data at a 10-meter resolution for comparative analysis. Regarding the overall accuracy (OA) and kappa coefficient, the results show that the OA reached 0.80 and that the kappa coefficient was 0.76. These results indicate that the supervised classification had a high reference value. To further analyze the forest cover, we extracted the forest part of the data from the supervised classification map. Data processing was performed via ArcMap software to convert the extracted forest raster data into point data, and each image element representing the forest was converted into a point. A total of 1,161,491 data points were generated (**Figure 5**), which provided detailed basic data for subsequent spatial analysis and model construction.

### 4.2 Sensitivity analysis of the vegetation indices

**Figure 6:**
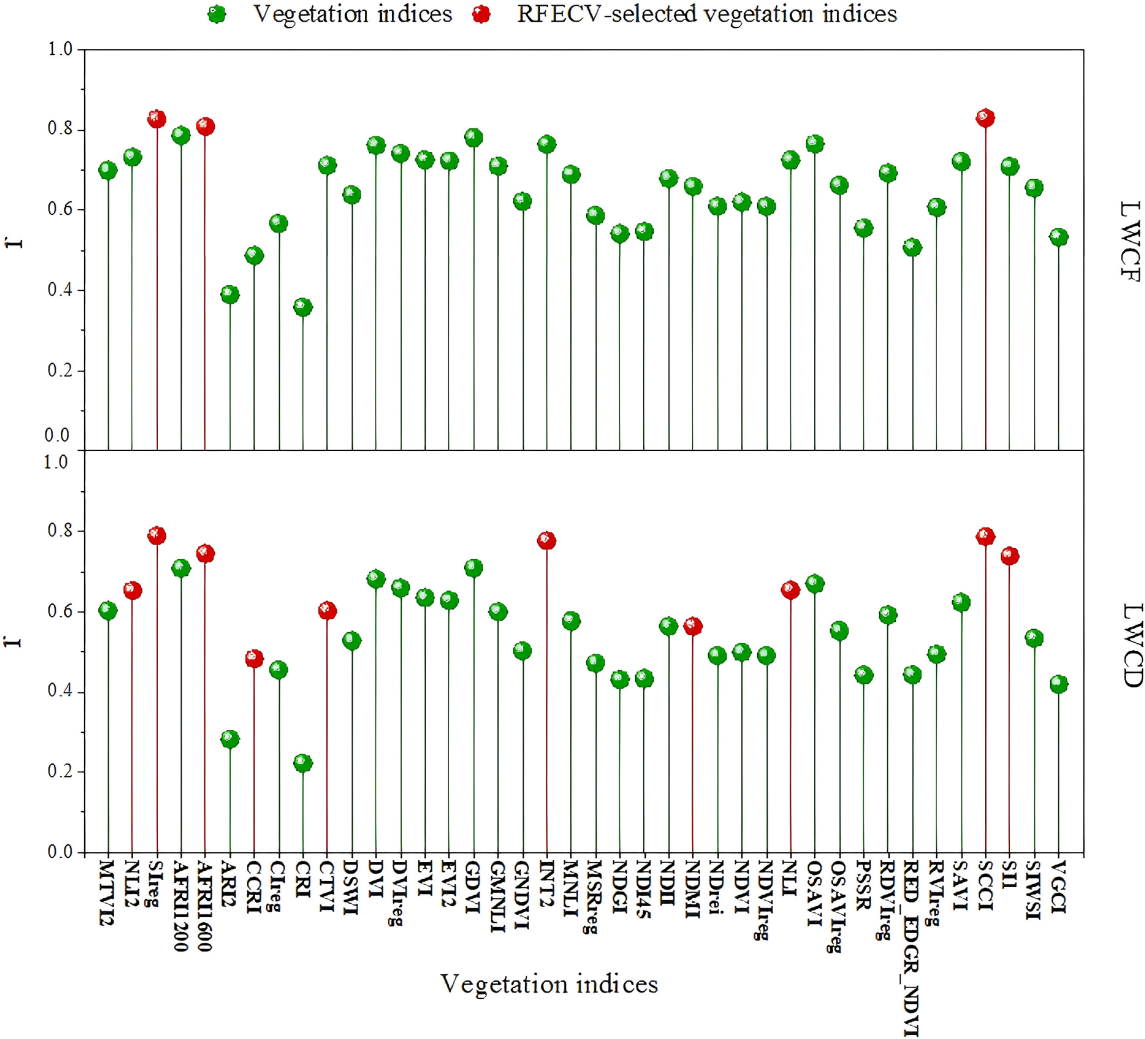
Correlation coefficients between the water content and vegetation indices and sensitive vegetation index selection.

The Pearson correlation coefficients (r) between these vegetation indices and LWCD and LWCF were calculated in this study (**Figure 6**). The results revealed that the r values of the correlation coefficients of 38 out of 40 vegetation indices were greater than 0.4 for the LWCF, indicating strong correlations; in particular, the r values of AFRI1600, SIreg, and SCCI were greater than 0.8, which demonstrated a very high correlation. However, the r values of ARI2 and CRI were lower than 0.4, indicating weak correlations. Similarly, the correlation coefficients between the LWCF and ARI2 and CRI were lower than 0.4, whereas the R values of the remaining 38 indices were greater than 0.4. In particular, the correlation coefficients of three indices, namely, AFRI1600, SIreg, and SCCI, were greater than 0.8, which indicated that these indices are strongly related to the dry-weight moisture content.

Overall, these 40 vegetation indices showed significant correlations with the needle leaf water content, with AFRI1600, SIreg, and SCCI displaying the highest correlations and associated with changes in the needle leaf water content. Therefore, these indices have high potential for application in monitoring changes in the needle leaf water content and recognizing signs of pest stress.

To further screen the vegetation indices that were most sensitive to the water content of needles, the RFECV method was used. In the analysis of LWCF, three multispectral vegetation indices, SIreg, AFRI1600, and SCCI, were ultimately screened as the most sensitive, whereas in the analysis of LWCD, a total of 10 multispectral vegetation indices, namely, NLI2, SIreg, AFRI1600, CCRI, CTVI, INT2, NDMI, NLI, SCCI, and SI1, were obtained through screening. On the basis of these final screened sensitive vegetation indices, a model was constructed to estimate the dry-weight water content and fresh-weight water content of EJD under stress.

### 4.3 Water content estimation model and accuracy evaluation

**Figure 7:**
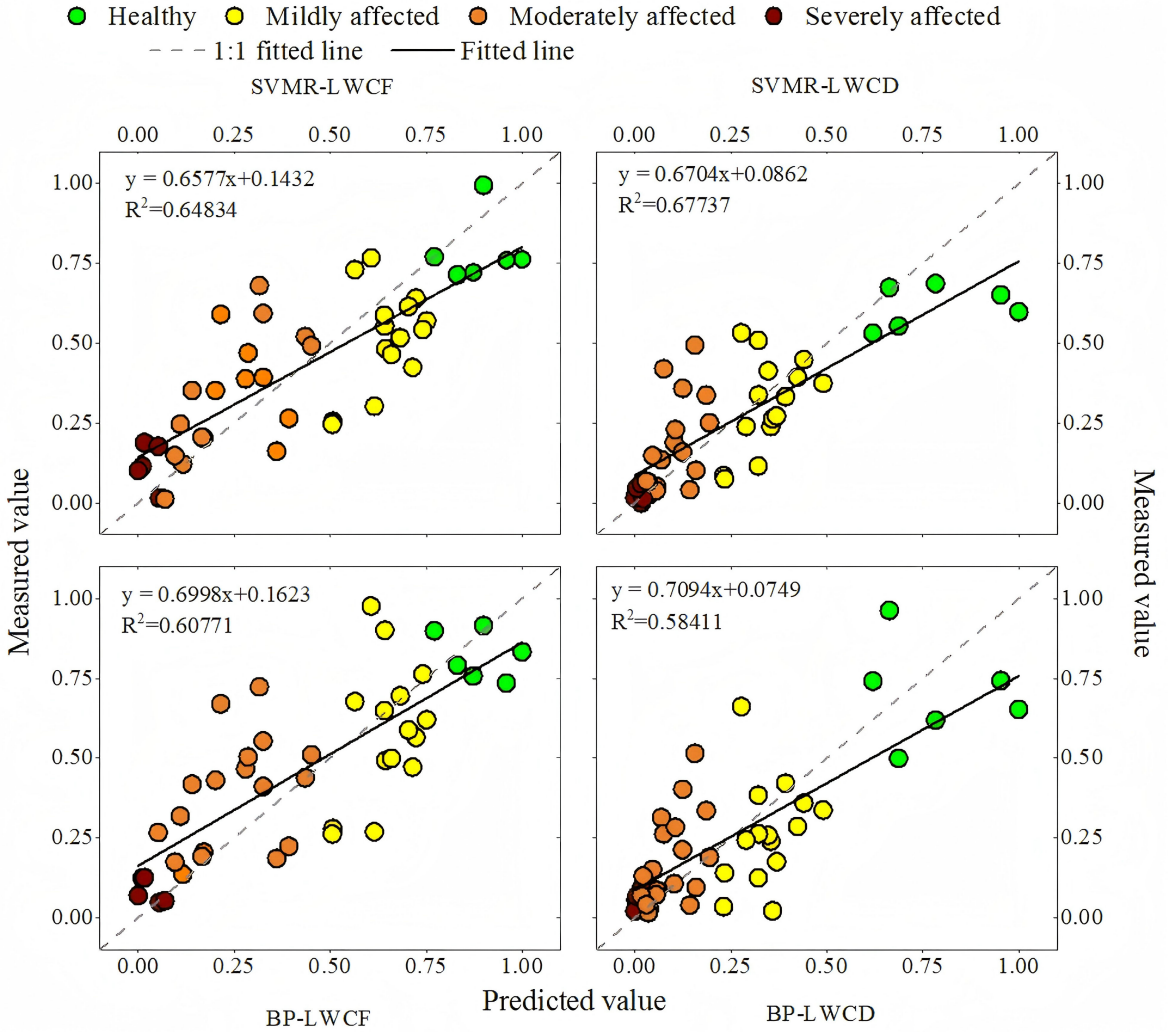
1:1 straight line fitting of predicted and measured values in the study area.

In this study, two water content estimation models, a BP neural network and an SVR model, were constructed on the basis of sensitive vegetation indices via MATLAB software, and the results of the performance evaluation are shown in **Table 3**. The LWCD-SVR model performed the best, with an MAE of 0.10507 and an RMSE of 0.14760, whereas the LWCF-BP model displayed comparatively lower accuracy, with an MAE of 0.15860 and an RMSE of 0.19549. To visualize the model fitting effect, a 1:1 fitting plot was created (**Figure 7**). On the basis of the performance of the two models under LWCF and LWCD conditions, the MAE and RMSE of the SVR model under LWCF conditions were better than those of the BP model, indicating its higher predictive performance. Under LWCD conditions, the models performed similarly, but the RMSE of SVR was slightly lower, indicating that it had an advantage in dry-weight moisture content estimation.

**Table 3:** Comparison of the estimation models’ accuracies.

Accuracy metrics Water content	BP		SVMR	
	MAE	RMSE	MAE	RMSE
LWCF	0.15860	0.19549	0.1477	0.17314
LWCD	0.13136	0.16831	0.10507	0.14760

From the modeling perspective, the R² values under LWCD conditions were better than those under LWCF conditions for both the SVR and BP models, indicating a good model fit in the prediction of the dry-weight moisture content. In particular, the SVR model displayed the most stable performance under LWCD conditions and was able to capture important data trends.

### 4.4 Spatial distribution of the water content

**Figure 8:**
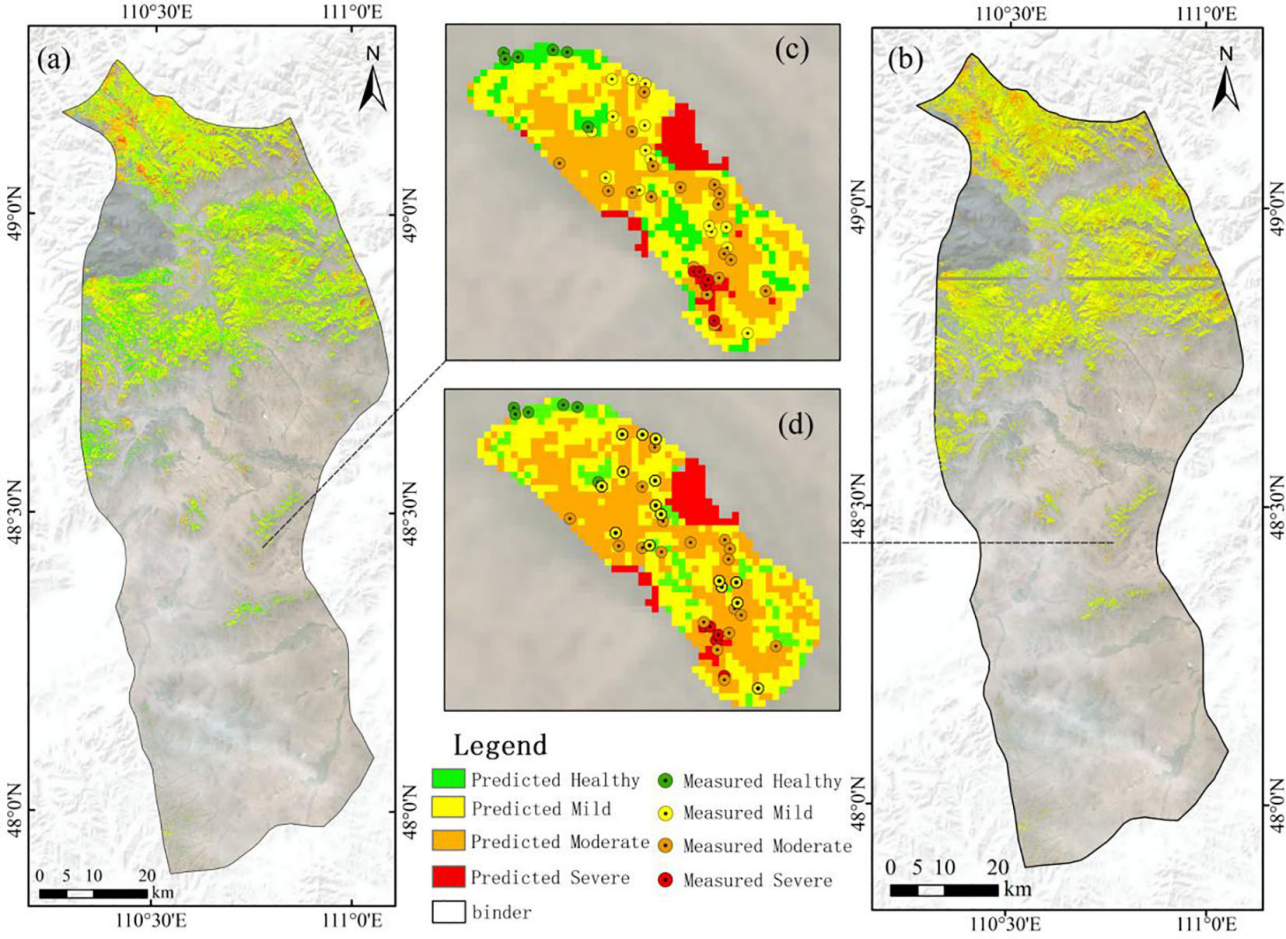
**(a)** Spatial distribution of the macroregional LWCF; **(b)** spatial distribution of the macroregional LWCD; **(c)** comparison of projected vs. actual values of LWCF in the study area; **(b)** comparison of projected vs. actual values of LWCD in the study area.

To visualize the water content of needles under the stress of EJD infection in the experimental area, in this study, we first performed the supervised classification of remote sensing images, extracted the forest area data, and finally acquired 1,161,491 forest pixel points via ArcMap software. For these extracted forest pixel points, we calculated the sensitive vegetation index features and applied them as input variables in the regression model that displayed the highest accuracy. By inputting these features into the SVR model, regression plots of the dry-weight moisture content and fresh-weight moisture content in the test area were generated. To further assess the health status of the needles, the moisture content range was categorized based on the degree of damage in the actual sampling data. Consequently, the estimated moisture content was classified into four levels: healthy (LWCF: >0.7; LWCD: >2.1), slightly damaged (LWCF: 0.5–0.7; LWCD: 0.6–2.1), moderately damaged (LWCF: 0.1–0.5; LWCD: 0.1–0.6), and severely damaged (LWCF:<0.1; LWCD:<0.1) (**Figure 8**).

In the visualized macroregional LWCF results, the central and southern regions are greenish in color, indicating that these regions generally contain healthy elements. In contrast, the northern region of Binder includes distinct orange and red areas, suggesting that the region is severely affected by pests. In addition, red pixels are scattered in the eastern and western regions, implying that there are some highly damaged elements in these regions. In **Figure 8c**, in the comparison of the actual damage with the predicted damage, two out of the six healthy samples were incorrectly predicted to be mild; four out of the 15 mild samples were predicted to be moderate; two out of the 18 moderate samples were predicted to be mild, and one was predicted to be severe; and two out of the five severe samples were predicted to be moderate.

In the visualized macroregional LWCD results, the overall color is skewed toward yellow, which indicates that most of the image elements in the region are only mildly affected. However, in the northern part of Binder, orange and red areas are evident, indicating that the region is severely affected. In addition, some red pixels are distributed in the eastern and western regions, indicating the presence of heavily damaged elements in these regions. In contrast, there are more healthy elements in the central and southern regions, indicating that the vegetation in this region is in relatively good condition. In **Figure 8d**, in the comparison of the actual damage level with the predicted damage level, only one out of the six healthy samples was incorrectly predicted to be mild; one out of the 15 mild samples was predicted to be moderate; four out of the 18 moderate samples were predicted to be mild; and three out of the five heavily damaged samples were predicted to be moderate.

### 4.5 Potential influence of the water content on the identification of larch stress by EJD

**Figure 9:**
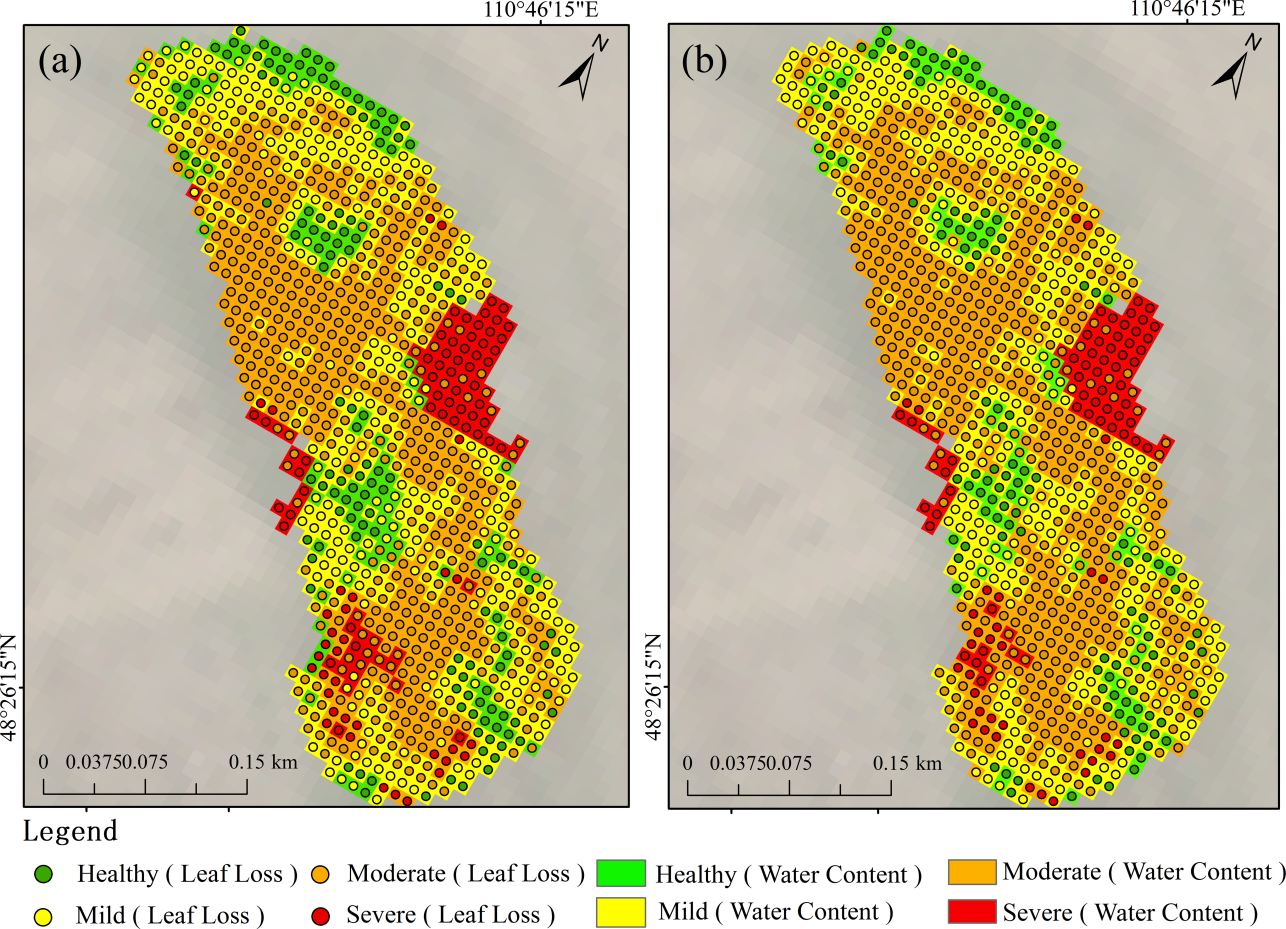
Comparative analysis of LLR classification and moisture content classification.

The LLR has been shown to be an effective indicator of tree damage and is often used to assess plant health. The LLR reflects changes in tree health under damage and stress conditions and is potentially correlated with changes in the water content of needles. Therefore, in this study, we selected the LLR in the field study area as an independent validation indicator to assess the accuracy of the needle leaf water content estimated on the basis of the vegetation indices. On the basis of 44 measured data points, we also estimated and categorized the LLR in the field study area and analyzed it in comparison with the categorized results of the vegetation index-estimated needle leaf water content, as shown in **Figure 9**.

The validation results indicate that the severity of leaf loss rate estimates in the field survey area shows a high degree of consistency with the severity of moisture content estimates, suggesting that leaf loss rate can effectively assist in validating the moisture content estimated through vegetation indices. Specifically, as shown in **Figure 9a**, the classification of LWCF and leaf loss rate is consistent across most areas. Notably, in moderately damaged areas, the performance of LWCF is particularly outstanding, with classification results aligning closely with the leaf loss rate. In healthy and lightly damaged areas, the classification results of LWCF and leaf loss rate are also relatively similar, indicating that fresh weight moisture content is a good reflection of tree health. However, in severely damaged areas, there are considerable discrepancies between the classifications of fresh weight moisture content and leaf loss rate. **Figure 9b** shows that the overall consistency between LWCD and leaf loss rate is slightly higher than that of LWCF, especially in healthy and lightly damaged areas, where the classification consistency is stronger. However, in moderately damaged areas, the performance of LWCD is slightly inferior to that of LWCF.

**Figure 10:**
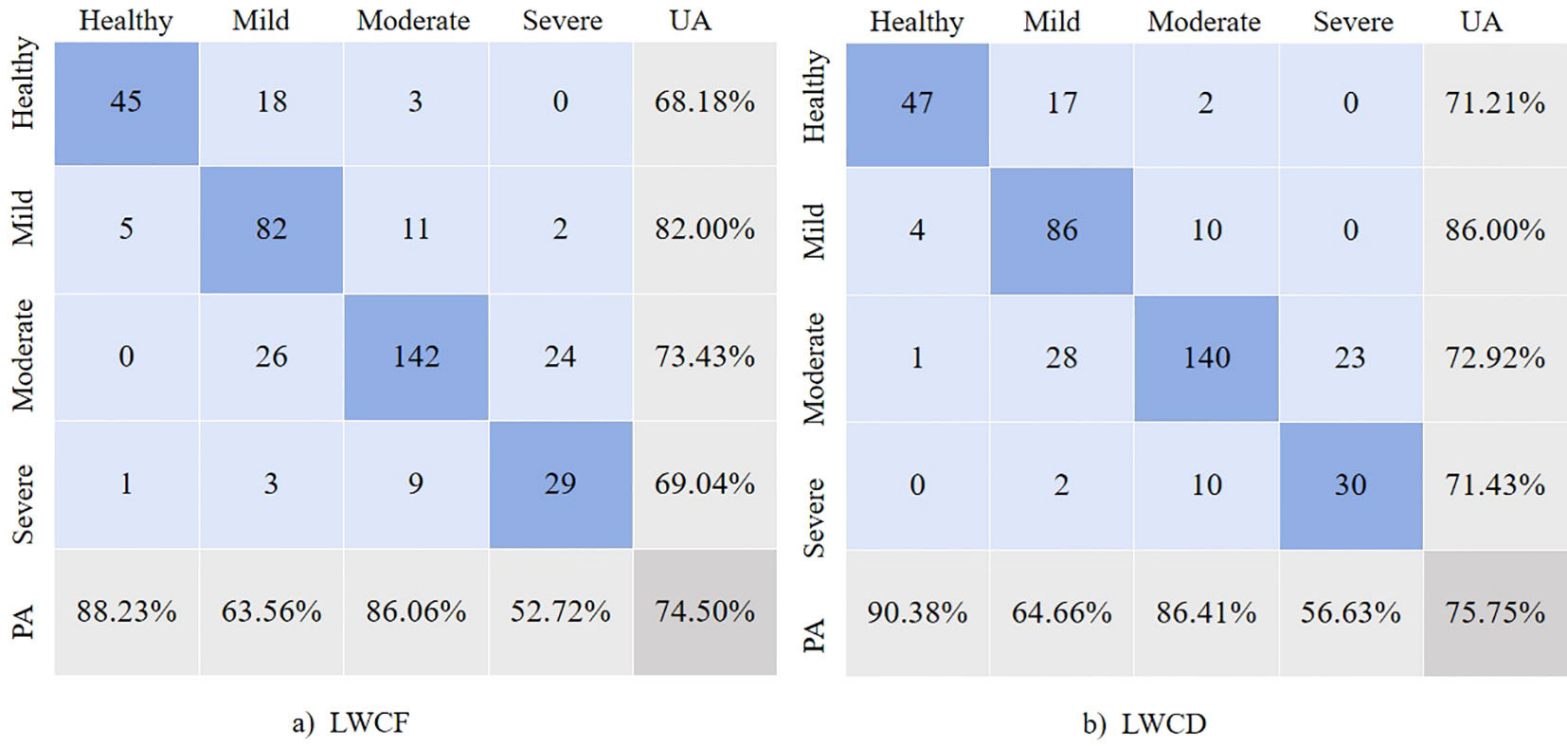
Confusion matrix for field-observed regions.

We randomly selected a subset of data from the field survey area and used the leaf loss rate classification as the ground truth to conduct a confusion matrix analysis (**Figure 10**). In the confusion matrix for LWCF, the OA was 74.50%, with a user accuracy (UA) of 68.18% for the healthy category and a producer accuracy (PA) of 88.23%. For the lightly damaged category, the UA was 82.00% and the PA was 63.56%. In the confusion matrix for LWCD, the OA was 75.75%, with a UA of 71.21% and a PA of 90.38% for the healthy category, and a UA of 86.00% and a PA of 64.66% for the lightly damaged category. These results indicate that the classification accuracy of LWCD is superior, especially in the healthy, lightly damaged, and severely damaged categories, where both UA and PA show improvement.

## 5 Discussion and conclusions

### 5.1 Sensitivity of vegetation indices

When larch is stressed by EJD, its internal biochemical components, such as water content, change significantly. This change produces different spectral responses in the visible and near-infrared bands, so the combination of these bands can be used to extract a vegetation index that reflects the health of the vegetation. In particular, when forest trees are stressed by insect pests, the response of each band is obvious, so the extraction of a sensitive vegetation index is especially critical because it can effectively reflect the changes in the water content of needles and leaves, thus providing a reliable basis for the estimation of the water content. In this work, 40 vegetation indices and Pearson values were calculated with respect to the water content, among which the most sensitive index was the SCCI, which can accurately capture vegetation health and changes by combining the near-infrared band information, red light band information, and NDVI. Under stresses caused by drought, pests, or diseases, the water content and chlorophyll content of coniferous trees decrease simultaneously. Owing to its sensitivity to chlorophyll changes, the SCCI can be used to detect water stress and reflect water content changes at an early stage and is therefore used for plant health monitoring and to provide early warnings of water stress. SIreg combines information from the blue band and the red-edge band to capture water content changes in conifers, especially B5, which plays a key role in monitoring the water content. When plants are subjected to water stress, the amount of intracellular water decreases, leading to changes in reflectance in the red-edge band. B2, although mainly used for other purposes, when combined with B5, can provide additional information regarding the photosynthesis and health status of plants under stress conditions. AFRI1600, which can be used to assess the moisture content of vegetation through the shortwave infrared band, can be combined with information from the near-infrared band to reflect the health status of vegetation. Therefore, this index is very sensitive to changes in the water content under drought conditions and can be effectively used for monitoring the moisture and stress levels of vegetation. These three indices were selected as sensitive vegetation indices to both LWCF and LWCD, and seven more indices were selected for the LWCD: NLI2, CCRI, CTVI, INT2, NDMI, NLI, and SI1. Among them, NLI2 is an enhanced vegetation index that reflects vegetation health on the basis of square information from the near-infrared band and the green light band (B3) to provide accurate assessments of the water content and photosynthesis efficiency of vegetation. The CCRI is mainly used to assess the chlorophyll concentration of plants. The ratio of the signal in the red light band to that in the red edge band can be used to assess the health of vegetation, especially under stress conditions, and the CCRI can be used to detect physiological changes in vegetation. The CTVI is an improved version of the NDVI designed to reduce noise and data fluctuations through the correction and transformation of NDVI values, especially in areas with low vegetation cover or under stress. By correcting and square-root transforming the NDVI, this index reflects the health of vegetation more consistently and shows greater robustness than the NDVI, especially when dealing with more complex vegetation or environmental conditions. INT2 is a composite vegetation index that captures the overall health of vegetation by combining the signals from the green, red, and red-edge bands and, in particular, reflects the chlorophyll content and water content. The NDMI is mainly based on information from the near-infrared band and the shortwave infrared band. B8 is used to reflect the cellular structure and water content of vegetation, and healthy vegetation is most reflective in the near-infrared band. B11 is used to identify changes in moisture content, especially soil moisture and vegetation water contents. The index can be used to effectively monitor changes in the moisture content of vegetation by comparing reflections in the near-infrared (NIR) and shortwave infrared (SWIR) bands. The NLI is mainly based on information from the near-infrared and red light bands, and through the non-linear computation of these two bands, it can be used to accurately assess the health of the vegetation and the chlorophyll content. In particular, the sensitivity to vegetation health is enhanced by the use of the square of the NIR band signal, which provides valuable information regarding photosynthetic efficiency and water content changes in vegetation. This non-linear combination is ideal for enhanced vegetation differentiation and health status assessment, such as in large-scale ecosystem monitoring and management. SI1 combines the red edge band and red light band signals and synthesizes the information from these two bands via square-root transformation; the result can then be used to assess the health status of vegetation.

In this study, the near-infrared band was used the most because it is very sensitive to changes in the moisture content, and healthy vegetation has a relatively high reflectance in the near-infrared band, whereas the reflectance decreases significantly under water stress. Thus, the NIR band plays a crucial role in monitoring plant water content and health. The second-most-important band is the shortwave infrared band, which is capable of capturing changes in the moisture content of vegetation, and B11 displays high sensitivity, especially when assessing moisture stress in vegetation. The shortwave infrared band is often combined with the near-infrared band and is commonly used for monitoring vegetation under drought and stress conditions. Additionally, the red light band reflects the chlorophyll absorption of vegetation. Healthy vegetation absorbs a large amount of red light, so the red band is ideal for assessing changes in chlorophyll content, especially when affected by stress, pests, or diseases, and the reflective properties of the red band can change significantly. Since the chlorophyll content is closely related to the water status of plants and water stress usually leads to a decrease in the chlorophyll level, the red light band can not only reflect changes in chlorophyll but also indirectly reflect changes in the water content. Therefore, the sensitivity of the red light band to chlorophyll makes it suitable for water content estimation. In addition, the red edge bands are spectral regions located between red and near-infrared light regions, and these bands are important for vegetation monitoring and are particularly sensitive to vegetation water content and chlorophyll concentrations. Changes in the cellular structure and water content of the leaves during vegetation growth significantly affect the red band reflectance. Finally, the green band is also used to monitor the health and growth status of vegetation by reflecting the chlorophyll content and photosynthetic efficiency. Healthy vegetation has specific reflectance properties in the green light band; thus, this band is useful for supplementing the estimation of water and chlorophyll levels.

In summary, the indices are based on combinations of different spectral features and can be used to assess vegetation health and water content changes from multiple perspectives, providing comprehensive and precise tools for vegetation monitoring under different environmental conditions. The selection and combination of these sensitive indices provide strong support for the water content prediction model in this study and improve the accuracy and stability of remote sensing-based monitoring.

### 5.2 Evaluation of estimation model accuracy

**Figure 11:**
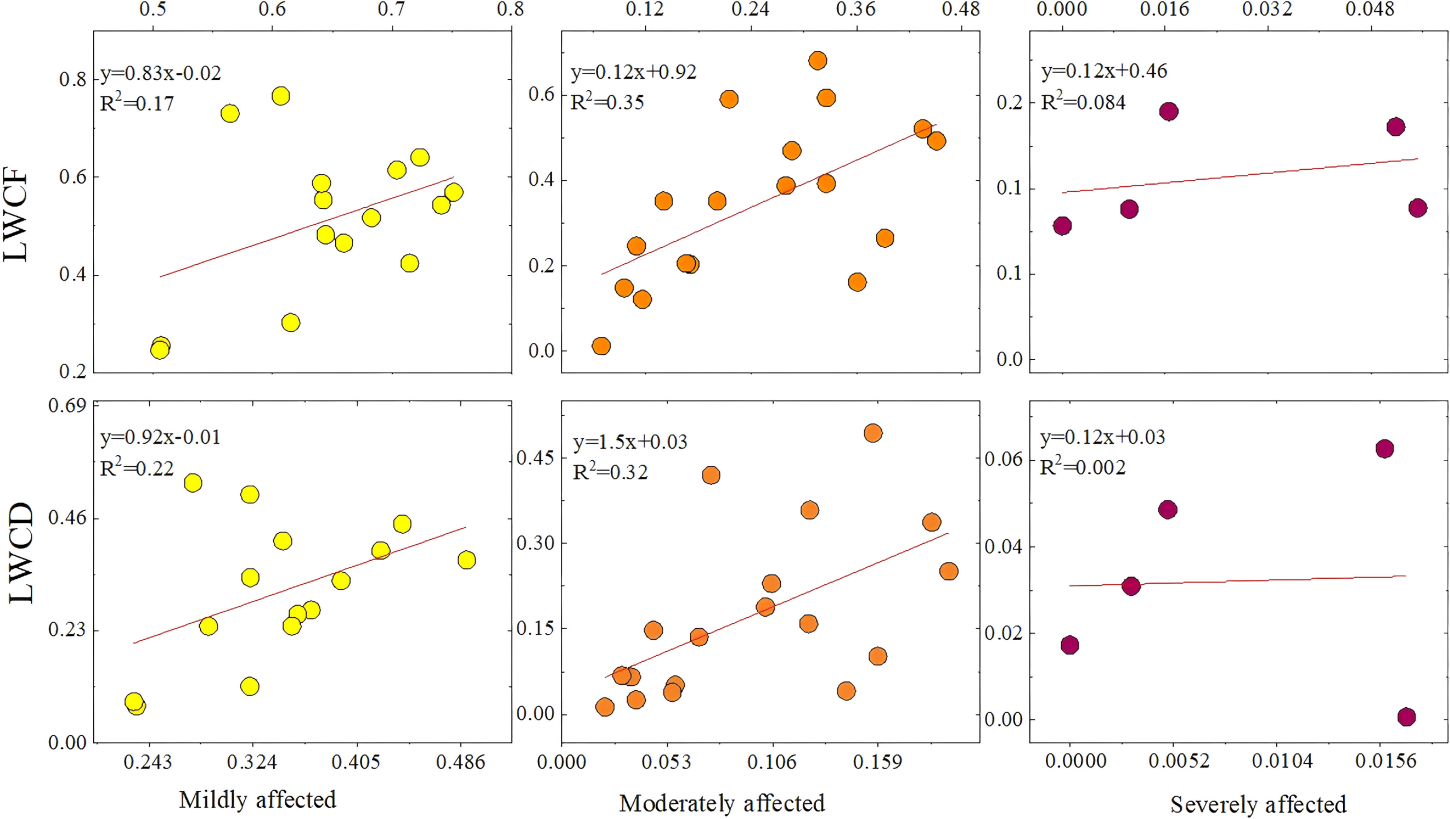
Scatterplots of mild, moderate, and severe damage predicted with the SVR model compared with the true values.

In this study, the SVR-predicted and measured values were fitted hierarchically into three categories, namely, mild, moderate, and severe, with the aim of accurately classifying the physiological status of plants with different degrees of damage. Compared with the overall fit, the graded fit reveals subtle differences in the regions of each level of damage, avoiding trend confusion and thus improving the analysis accuracy. Scatter plots revealed that the LWCF and LWCD exhibited different trends in the mild, moderate, and severe damage regions. The LWCD results were better than those for LWCF in mildly damaged areas, whereas in moderately damaged areas, the LWCF was more sensitive than the LWCD and better able to capture plant health. However, the correlation between the LWCF and LWCD was significantly weaker in heavily damaged regions, likely because plant physiological changes were extreme in these areas, resulting in small fluctuations in the water content and insufficient information for differentiating the degree of damage. In addition, there were fewer data points in the heavily damaged areas, and the insufficiency of samples increased the volatility of the regression analysis results, further affecting the fitting effect. Therefore, reducing the number of data points is an important factor contributing to the instability of regression analysis results in heavily damaged regions. This suggests that the performance of the model is limited by the number of samples and the sensitivity of physiological indicators in heavily affected regions (**Figure 11**).

In this study, the LWCF and LWCD were also estimated at a large scale via SVR, and their spatial distributions were obtained. The LWCF is the water content of the plant in its natural state and is based on the ratio of the water content to the total weight of the plant when it is fresh. This metric can accurately reflect the immediate physiological state of plants under well-watered conditions and is directly related to photosynthesis, stomatal regulation, and the water evaporation rate of plants. When a plant suffers from insect damage, its water regulatory mechanism and leaf health are immediately affected, and changes in the LWCF can sensitively reflect the water status of the plant; thus, the health trend of the plant can be predicted. By monitoring changes in the LWCF, it is possible to assess whether plants are able to maintain their growth via normal photosynthesis and transpiration in the short term. This analysis aids in the development of effective management and protective measures to ensure plant survival under pest stress. **Table 3** shows that the MAE and RMSE of the SVR model were also slightly lower than those of the BP model in the prediction of the LWCF, indicating that the SVR model is superior for predicting the fresh-weight water content. **Figure 7** also shows that the SVR model yields a better fit than the BP model. On the basis of the LWCF fitting graph, for healthy samples, the fitting results for light and medium damage are better and the results for heavy damage, as also illustrated in **Figure 8**. Moreover, most of the areas in Binder are healthy or mildly affected, indicating that photosynthesis and transpiration are normal in most areas in the short term, resulting in sustained growth. The figure also shows that many areas in Binder are mildly damaged, with outbreak areas evident in the north, east, and west.

The LWCD is the proportion of water contained in the dry matter remaining after the plant has been completely dehydrated (e.g., by drying and other processes); it reflects a plant’s ability to hold water under water deficit conditions and is an important indicator for assessing plant health and drought tolerance. When plants are subjected to insect pests, their water regulation ability and leaf health are strongly affected, and changes in the LWCD can directly reflect the physiological status of plants under insect pest stress. By monitoring the LWCD, changes in plant health status can be recognized in a timely manner, providing a scientific basis for the development of pest control strategies and reducing the degree of ecological damage caused by pests. **Table 3** shows that in the prediction of the LWCD, the SVR model performed better than the BP model did, and the MAE and RMSE of the SVR model were lower than those of the BP model, which indicates that the SVR model yields better prediction accuracy in estimating the dry-weight water content. **Figure 7** also shows that the SVR model yields a better fit than the BP model does. On the basis of the fitting graph of the LWCD, the overall fit is better than that for the LWCF, but there are some problems in the heavily damaged area. In particular, some of the heavily damaged data points are close to moderately damaged data points, making it difficult to clearly distinguish between them. This may be because the range of variation in dry-weight moisture content is not limited to the interval of 0–1. Notably, the data were normalized for presentation. Since normalization compresses the range of true values of dry-weight moisture content, it results in heavy and medium damage values being similar, thus creating spatial overlap in the fitting results. In addition, even in the non-normalized case, the small magnitude of water content changes and the tendency for physiological responses to be similar in the heavily and moderately affected regions results in overlapping data regarding the needle and leaf water contents, further increasing the difficulty of classification, as shown in **Figure 8**. Moderate and severe damage is evident in the north, east, and west, which is consistent with the LWCF results.

The results in **Figure 8** are consistent with the findings in **Figure 7**, further validating the prediction ability of the model under different stress levels. The model performs best in healthy, light, and moderate damage cases, and there is some error in the predictions for areas of heavy damage. The above analysis provides a basis for improving the model.

### 5.3 Comparison analysis of leaf loss rate and leaf water content

In this study, the high degree of consistency between the leaf loss rate and leaf water content estimation results validates the effectiveness of remote sensing data in forest health monitoring. Notably, in moderately damaged areas, the classification results of LWCF closely align with the leaf loss rate, indicating that fresh weight moisture content effectively reflects tree health. However, in severely damaged areas, significant misclassifications were observed, especially where the classification results of both LWCF and LWCD failed to accurately identify these areas. This discrepancy may be attributed to the greater variability of fresh weight moisture content in severely damaged areas, which leads to a more ambiguous signal in the remote sensing data, thus affecting classification accuracy.

In the confusion matrix analysis, the overall consistency of LWCD was slightly higher than that of LWCF, particularly in healthy and lightly damaged areas, where it demonstrated higher classification accuracy. However, in moderately damaged areas, the performance of LWCD was slightly inferior to LWCF, suggesting that dry weight moisture content is less effective in identifying moderately damaged areas. For severely damaged areas, both LWCF and LWCD showed misclassifications, particularly where some severely damaged areas were misclassified as lightly or moderately damaged. This indicates that both moisture content estimation methods still require improvement in their ability to adapt to extreme damage zones.

Although the LWCD yielded better results than the LWCF, importantly, both performed relatively well in classifying areas of light and moderate damage. These findings suggest that the use of either the LWCD or the LWCF can provide effective support for monitoring larch looper. Accurate identification of light damage provides a critical basis for early monitoring, which can aid in establishing timely preventive and management measures to avoid further deterioration. Accurate identification of moderate damage can effectively reflect the physiological changes in plants under stress, providing a scientific basis for the development of targeted restoration and protection strategies.

### 5.4 Conclusion

In this study, we used UAV and remote sensing data combined with field measurement data to select sensitive vegetation indices for the LWCD and LWCF in larch under geometrid stress via RFECV, constructed effective LWCD and LWCF estimation models via SVR and BP methods, and mapped large-scale regional visualizations based on the LWCD and LWCF classification models with the highest accuracy. The conclusions are as follows:

1. When the LWCD and LWCF were estimated via the SVR and BP models, the SVR model yielded higher accuracy and stability.
2. The high classification accuracy of the LWCD and LWCF in mildly and moderately infested areas suggests that they can be used as effective indicators for assessing EJD abundance to assist in monitoring the occurrence and development of infestations.
3. Through model accuracy assessment and auxiliary validation, the prediction results show that the LWCD-based results are more stable and reliable in terms of performance and have greater credibility than the LWCF results.

In this work, we provide high-precision estimation models for the LWCD and LWCF under larch looper stress conditions, thus providing reliable technical support for the early monitoring of this pest. By establishing such an early warning system, monitoring methods based on the LWCD and LWCF can help forestry departments recognize and respond to potential pest risks in a timely manner, providing a scientific basis for protecting the environment and guaranteeing the sustainable development of forestry resources.

## Data Availability

The original contributions presented in the study are included in the article/supplementary material. Further inquiries can be directed to the corresponding author.
